# Clinical significance of the ‘not otherwise specified’ subtype in candidates for resectable non-small cell lung cancer

**DOI:** 10.3892/ol.2014.2302

**Published:** 2014-07-01

**Authors:** SHINYA TANE, WATARU NISHIO, HIROYUKI OGAWA, DAISUKE HOKKA, KENTA TANE, YUGO TANAKA, SHUNSUKE TAUCHI, KAZUYA UCHINO, YASUHIRO SAKAI, CHIHO OHBAYASHI, MASAHIRO YOSHIMURA, YOSHIMASA MANIWA

**Affiliations:** 1 Division of Thoracic Surgery, Kobe University Graduate School of Medicine, Kobe, Hyogo 650-0017, Japan; 2 Department of Thoracic Surgery, Hyogo Cancer Center, Akashi, Hyogo 673-8558, Japan; 3 Division of Pathology, Kobe University Graduate School of Medicine, Kobe, Hyogo 650-0017, Japan; 4 Division of Pathology, Nara Medical University, Kashihara, Nara 634-8521, Japan

**Keywords:** non-small cell lung cancer, not otherwise specified, transbronchial biopsy, histological subtypes, prognostic factor, surgery

## Abstract

The histological subtype of non-small-cell lung cancer (NSCLC) is a significant factor when selecting treatment strategies. However, cases are occasionally encountered that are diagnosed as ‘not otherwise specified’ (NOS) prior to surgery, due to an uncertain histological subtype. The present study investigated the prognostic significance of the NOS subtype for patients with resectable NSCLC. Between 2001 and 2011, 1,913 patients were diagnosed with NSCLC using transbronchial biopsy and underwent surgical resection at two facilities in Japan. Of these patients, 151 (7.9%) were pre-operatively diagnosed with NSCLC-NOS (NOS group) and the remainder had confirmed histological subtypes (confirmed group). The present study compared the clinicopathological features and prognoses of these groups. Analyses of resected specimens revealed that pleomorphic cell carcinoma, large cell neuroendocrine cell carcinoma, large cell carcinoma and adenosquamous carcinoma were significantly more common in the NOS group than in the confirmed group (P<0.001, P=0.002, P=0.019 and P=0.014, respectively). The five-year survival rate was significantly poorer in the NOS group (60.5 vs. 67.1%; P=0.010), particularly for stage I disease (70.8 vs. 80.7%; P=0.007). The results of a multivariate analysis of overall survival indicated that NOS was a significant independent prognostic factor (hazard ratio, 1.40; 95% confidence interval, 1.02–1.86; P=0.041). These results indicated that pre-operative NOS was significantly associated with poorer survival, including for stage I disease. In conjunction with other clinicopathological parameters, NOS can be a useful prognostic factor when deciding on a treatment strategy for NSCLC.

## Introduction

Lung cancer is a leading cause of cancer-related mortality worldwide. From a histological perspective, the field of lung cancer treatment has been relatively static for several decades. Yet, several studies have shown that the histological subtyping of non-small cell lung cancer (NSCLC) is extremely important in predicting response rates, progression-free survival and specific drugs toxicities (1–3). For example, NSCLC subtypes differ significantly with respect to the prevalence of specific molecular alterations, including the epidermal growth factor receptor (EGFR) gene (3). Consequently, treatments are selected according to the histological subtypes of NSCLC on a daily basis.

Although novel diagnostic procedures, improved imaging modalities and new immunostaining techniques have improved histological accuracy, pathological examination occasionally fails to subtype NSCLC, leading to the rather non-specific diagnosis of NSCLC not otherwise specified (NOS). NOS diagnoses are often the consequence of small sample sizes and highly heterogeneous tumors, which limit the consistency and accuracy of subtyping using bronchoscopic biopsies. It has been reported that NOS is an unfavorable independent prognostic factor among stage IV NSCLCs, as NOS is associated with an aggressive tumor biology (4). However, the prognostic value of NOS in resectable NSCLC has not been studied.

Thoracic surgeons typically select from among the available surgical procedures according to the lung tumor type. For instance, limited resection has recently been recommended for early lung cancer that is peripherally located and exhibits a glass ground opacity (associated with minimum invasive adenocarcinoma) on thin-section computed tomography (CT) (5). Conversely, limited resection may be inappropriate as a curative surgery for certain aggressive tumors, even those that are small (6). The NOS subtype is occasionally encountered pre-operatively, yet no surgical consensus has been established for NOS tumors.

Therefore, the present study sought to assess the association between a pre-operative NOS subtype and the prognosis of candidates for resectable NSCLC. Accordingly, this study aimed to retrospectively determine whether pre-operative NOS can provide prognostic information for patients who undergo surgical resection for NSCLC. Additionally, the study sought to clarify the association between a pre-operative NOS classification and the pathological features of the resected specimen.

## Materials and methods

### Patients

The clinical data of 2,519 patients with primary NSCLC who underwent complete surgical resection at the Kobe University Hospital and Hyogo Cancer Center (Kobe, Hyogo, Japan) between January 2001 and December 2011 was retrospectively examined. In total, 20 patients were excluded due to incomplete data. Of the 2,499 remaining patients, 2,309 had undergone a pre-operative bronchoscopy to establish the tumor malignancy, and 1,913 of these were diagnosed with NSCLC (396 patients were excluded due to pre-operative biopsy results that were ‘negative’ or ‘suspicious’ for malignancy). The 1,913 patients included in the present study were divided into two groups: Cases diagnosed as NOS (the NOS group) and cases with confirmed specific histological subtypes (the confirmed group), and their clinical features and outcomes were compared. The Kobe University Hospital and Hyogo Cancer Center institutional review boards approved the study and each participant provided informed consent. All patients were operated on with curative intent. The candidates for limited resection, such as segmentectomy and wedge resection, were selected by the judgment of the surgeon responsible, who considered resectability and the ability to obtain enough surgical margins from the tumor. Patients with salivary gland-type tumors, carcinoids and small cell carcinoma were excluded. All patients treated with induction therapy were also excluded. Medical records provided data on patient age, gender, body mass index (BMI), smoking status, respiratory function, stage, surgery, pathological findings, adjuvant therapy and prognosis. Contrast-enhanced CT scans of the chest, abdomen and head, bone scintigraphy and positron emission-CT since 2006 were executed routinely for pre-operative evaluation. Staging was determined according to the new International Union Against Cancer Staging System (7).

### Diagnostic techniques

During the bronchoscopy sessions, cytological and histological diagnostic procedures were performed whenever feasible. The diagnostic results of cytological materials (transbronchial needle aspiration or transbronchial brushing cytology) were obtained for all cases. Histological diagnosis (bronchoscopic biopsy) was performed wherever sufficient tumor tissue material was available. If necessary, immunostaining was also performed to maximize the diagnostic accuracy using biopsy material.

### Sample analysis

All samples were reviewed by two expert pathologists. Carcinomas diagnosed using pre-operative transbronchial samples were classified as adenocarcinomas, squamous cell carcinomas or NSCLC-NOS depending on the cytological diagnosis. These carcinomas were also classified as adenocarcinomas, squamous cell carcinomas, large cell carcinomas, combined tumors, adenosquamous cell carcinomas or NSCLC-NOS by histological examination. Surgical specimens were morphologically classified according to the 2004 World Health Organization classification criteria (8).

### Representative case

**Figure 1:**
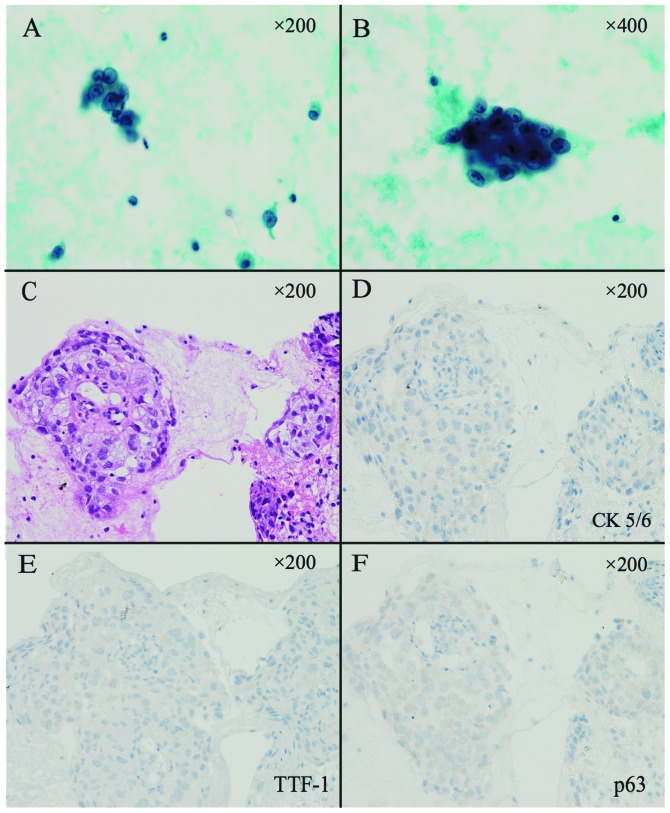
Representative case of diagnosed NOS. Cytological and histological examinations resulted in the classification of NSCLC-NOS due to a poorly-differentiated subtype (A and B) Papanicolaou staining of cytological specimens. (C) Hematoxylin and eosin staining of biopsy specimens. Immunohistochemistry was negative for (D) cytokeratin (CK) 5/6, (E) thyroid transcription factor-1 (TTF-1) and (F) p63, indicating that there was no differentiation toward adenocarcinoma or squamous cell carcinoma. NOS, not otherwise specified.

A representative NOS case is shown in Fig. 1. As this case was subtyped as NSCLC-NOS according to bronchial smear and biopsy material, immunohistochemistory (IHC) was additionally performed. The IHC results were considered to indicate NSCLC-NOS if they included negative findings for thyroid transcription factor-1 (TTF-1), cytokeratin (CK)5/6 and p63 (9). The majority of pulmonary adenocarcinomas expressed TTF-1, whereas the majority of squamous cell carcinomas expressed CK5/6 and p63.

### Follow-up

Post-operative follow-up generally proceeded as follows. During the 2 years after surgical intervention, systemic and local examinations were performed every six months, including blood tests, chest and abdominal CT, magnetic resonance imaging and bone scintigrams. Between three and five years post-surgery, these intensive examinations were performed every year. To check for tumor recurrence and determine survival, observational follow-up was continued indefinitely or for at least five years.

### Statistical analysis

Statistical analyses were performed using JMP software, version 8 (SAS Institute, Cary, NC, USA). Differences between the NOS and confirmed groups were analyzed using Student’s t-test and the χ^2^ test with regard to gender, age, BMI, smoking status, respiratory function, size of tumor, surgical procedure, histological subtype and pathological stage between the NOS and confirmed groups. With respect to surgical procedures, segmentectomy and wedge resection were considered to be limited resection. The duration of overall survival was defined as the interval between the day of the surgery and the date of mortality (by any cause) or the last recorded follow-up. Disease-free survival was defined as the interval between resection and the proven detection of recurrence or metastases. Disease-free survival and overall survival were estimated using the Kaplan-Meier method, and differences in survival distributions were evaluated using the log-rank test. The Cox proportional hazards model was used to evaluate the association between the prognostic factors and survival rate following pulmonary resection, in terms of hazards ratios and 95% confidence intervals. P<0.05 was used to indicate a statistically significant difference.

## Results

### Included patients

**Figure 2:**
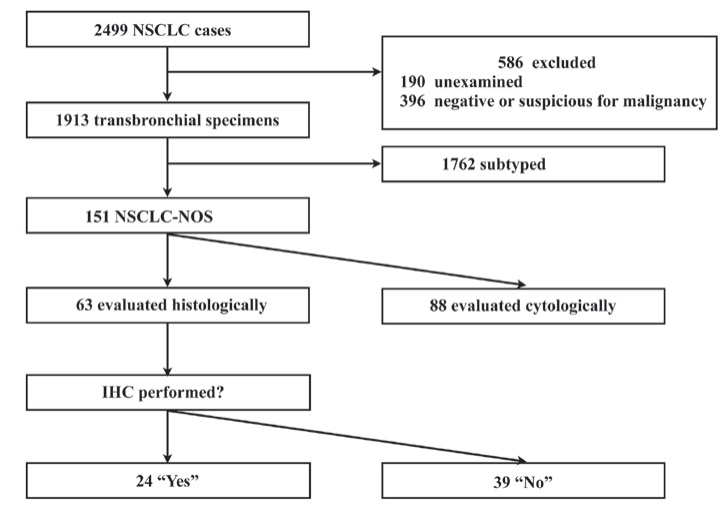
Flow chart of the inclusion of patients. NSCLC, non-small cell lung cancer; NOS, not otherwise specified; IHC, immunohistochemistry.

Of 2,519 patients with primary NSCLC who underwent complete surgical resection between January 2001 and December 2011, 1,913 satisfied the inclusion criteria. Fig. 2 presents a flow chart of the inclusion and exclusion criteria and diagnostic procedures. The initial sample included 1,662 males and 837 females, with a median age of 69 years (range, 30–91 years). Resected tumors were 3–170 mm in size (median, 28 mm).

### Diagnosis of NOS

Of the included cases, 151 (7.9%) were pre-operatively diagnosed as NOS. Table I presents the association between NOS findings and clinicosurgical factors. The NOS subtype was more frequently observed among male patients, smokers and patients with chronic obstructive pulmonary disease (COPD). In total, 57 (37.7%) patients received adjuvant chemotherapy in the NOS group, whereas 502 (28.4%) patients received adjuvant chemotherapy in the confirmed group.

A total of 88 NOS cases (58.3%) were diagnosed using cytomorphology alone. The remaining 63 NOS cases were evaluated histologically. IHC was performed in 24 of the histologically evaluated cases.

### Tumor histology and staging

Table II presents the distribution of histologies and pathological stages in the NOS and confirmed groups. In the NOS group, the histopathological types were ultimately determined on the basis of the resected specimens; 60 (39.7%) adenocarcinomas, 42 (27.8%) squamous cell carcinomas, 19 (12.6%) pleomorphic cell carcinomas, 12 (7.9%) large cell neuroendocrine cell carcinomas, 8 (5.3%) adenosquamous carcinomas, 8(5.3%) large cell carcinomas and 2 (1.3%) sarcomatoid carcinomas. Pleomorphic cell carcinoma, large cell neuroendocrine cell carcinoma, large cell carcinoma and adenosquamous carcinoma were significantly more common in the NOS group than in the confirmed group (P<0.001, P=0.002, P=0.019 and P=0.014, respectively). The NOS group included 39 (25.8%) stage IA, 51 (33.8%) stage IB, 24 (15.9%) stage IIA, 14 (9.3%) stage IIB and 23 (15.2%) stage IIIA cases. No NOS cases were stages IIIB or IV. The pathological stage distribution did not differ significantly between the NOS and confirmed groups (P=0.127).

### Follow-up and overall survival

**Figure 3:**
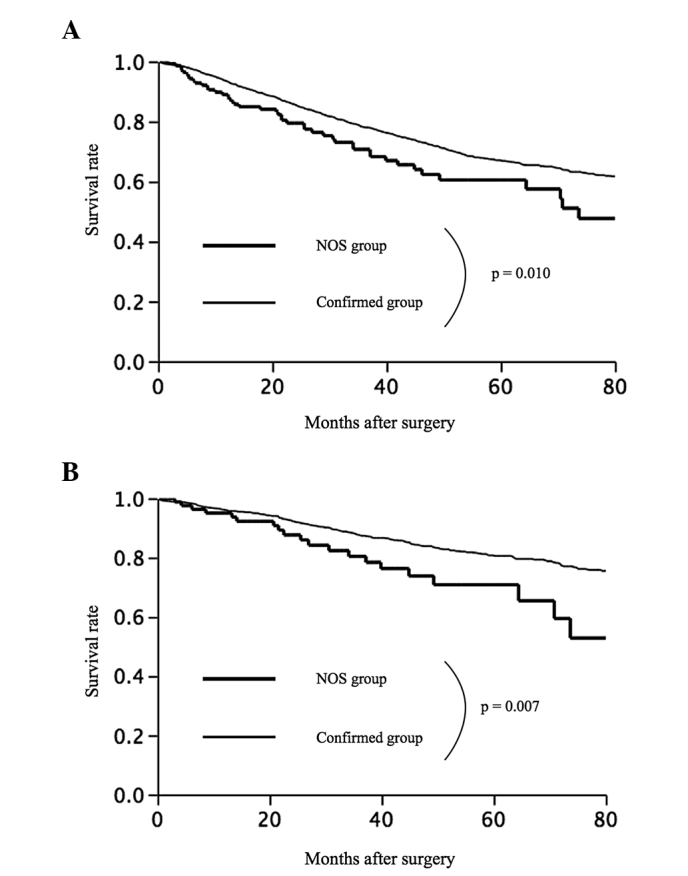
(A) Comparison of overall survival for patients with not otherwise specified (NOS) and confirmed subtype non-small cell lung cancer (NSCLC). (B) Comparison of overall survival for patients with NOS and confirmed subtype NSCLC of pathological stage I.

Of the 1,370 patients in the study who were not known to have succumbed, 908 (66.2%) were lost to follow-up during the initial five-year post-operative periods, and the remaining 462 (33.8%) were followed up for over five years. In the group of patients that remained (NOS and confirmed groups), the median duration of follow-up was 40.8 months (range, 0.4–145 months). Fig. 3 summarizes the overall survival rates observed in the study. The five-year survival rates were 60.5% in the NOS group and 67.1% in the confirmed group (Fig. 3A). Overall survival was significantly poorer in the NOS group than in the confirmed group (P=0.010). Among the 1,168 patients with stage I disease, the five-year survival rates were 70.8% in the NOS group and 80.7% in the confirmed group (P=0.007) (Fig. 3B).

### Disease-free survival

**Figure 4:**
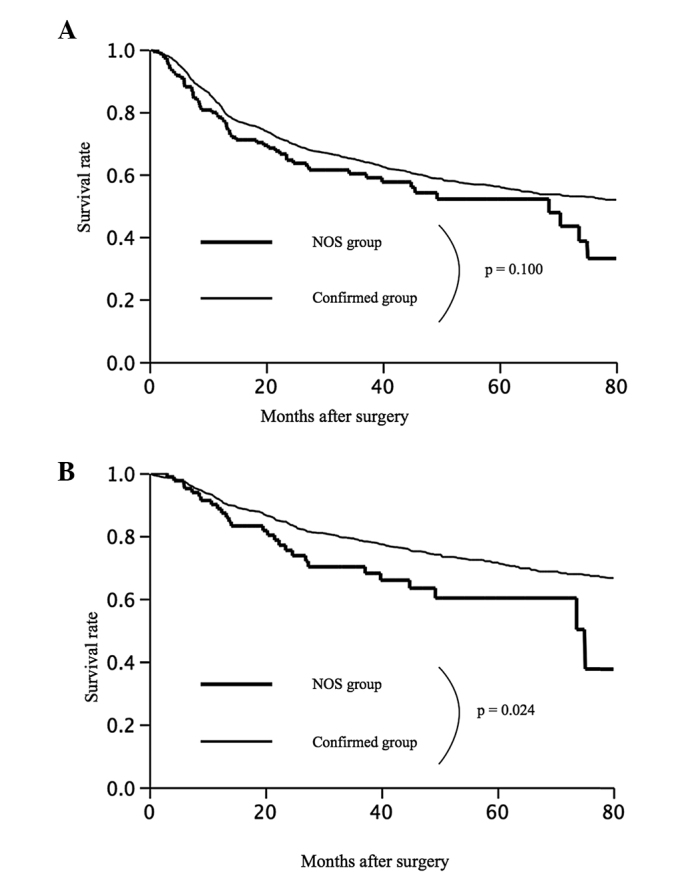
(A) Comparison of disease-free survival for patients with not otherwise specified (NOS) and confirmed subtype non-small cell lung cancer (NSCLC). (B) Comparison of disease-free survival for patients with NOS and confirmed subtype NSCLC of pathological stage I.

The five-year disease-free survival rates were 52.1% in the NOS group and 60.0% in the confirmed group (P=0.100) (Fig. 4A). The disease-free survival rate did not significantly differ between these two groups, but tended to be worse in the NOS group. Among the patients with stage I disease, the five-year survival rates were 71.3% in the NOS group and 60.2% in the confirmed group (P=0.020) (Fig. 4B).

### Subtyping

To assign cases to the NOS subtype, cytological and histological methods were relied upon. The association between the different diagnostic methods and the survival differences were analyzed in order to determine any correlations. Cytologically diagnosed NOS cases (n=88) exhibited a 65% five-year survival rate, whereas histologically diagnosed NOS cases (n=63) exhibited a 50.4% five-year survival rate, but this difference was not significant (P=0.378).

### Clinical variables

Additionally investigations were made into the associations between the clinical variables and overall survival in the total population (Table III). According to univariate analyses, NOS, age, gender, BMI, pathological stage, COPD, smoking status, histology, vessel invasion, lymphatic invasion and pleural invasion were each significantly associated with post-operative prognosis. Multivariate Cox regression analysis indicated that NOS, age, gender, BMI, pathological stage, histology, vessel invasion, lymphatic invasion and pleural invasion were independent prognostic factors in all the tested patients.

## Discussion

As novel, molecular targeted agents have type-specific efficacy and adverse effects, accurate identification of the primary lung cancer type is a necessity. For example, among patients with lung cancer who are treated with bevacizumab, those with squamous cell carcinoma are at increased risk from life-threatening hemorrhage (1). A recent study showed that combined cisplatin and pemetrexed treatment resulted in statistically greater survival rates compared with combined cisplatin and gemcitabine, but only for adenocarcinomas and large cell carcinomas (not for squamous cell carcinomas) (2). Moreover, the response to the EGFR-tyrosine kinase inhibitors, gefitinib and erlotinib, is strongly associated with the adenocarcinoma subtype (3). These studies pioneered the use of the histological subtypes as key determinants of treatment strategies for advanced NSCLC. The most current Multidisciplinary Classification of Lung Adenocarcinoma, jointly issued by the International Association for the Study of Lung Cancer, the American Thoracic Society and the European Respiratory Society, recommends that NOS be assigned as infrequently as possible (10,11). However, a NOS classification is unavoidable in specific cases, as routine morphology and immunohistochemistry cannot differentiate certain tumor cells. Sigel *et al* (12) found that NOS was diagnosed in 12% of cytology and 6% of biopsy specimens. Where paired specimens were available (representing the two methods), the prevalence of NOS decreased to 4%. In the present study, it was found that 7.9% of cases were classified as NOS, a rate comparable to that previously reported (4,12,13).

NOS is generally diagnosed using cytology or biopsy specimens, and not by surgically resected specimens. For the cases of advanced-stage NSCLC, resected specimens were unavailable in the present study. Consequently, the true histology or correlation between the histological subtypes and the prognosis of the NOS patients could not be determined. Therefore, the study was limited to the resected cases. To the best of our knowledge, the present study is the first to examine whether pre-operative NOS can provide prognostic information for patients who undergo surgical resection for NSCLC.

We hypothesize that there are two principal causes of a NOS diagnosis. First is the nature of the biopsy itself; it can be difficult to obtain more than a scant bronchial specimen, which lacks distinctive features. In the present study, all transbronchial procedures were performed using a conventional bronchoscope under radiographic guidance. However, several recent studies have indicated that endobronchial ultrasound-guided transbronchial biopsy (EBUS-TBNA) is a widely accepted method for diagnosing thoracic tumors (14,15). The EBUS-TBNA scope can be used to assess and diagnose intrapulmonary lesions not visible through a conventional bronchoscope, as long as they are within the reach of the EBUS-TBNA scope. Consequently, EBUS-TBNA provides relatively high yields for diagnosing lung tumors. However, the EBUS-TBNA scope and other novel devices often fail to recover tumoral specimens if the tumor is located in the peripheral lung parenchyma or if the tumor interior is necrotic. By excluding the 396 (15.7%) cases of suspicious and negative results in the present study, the effect of the variations in transbronchial procedure was minimized.

Second, the NOS subtype may be assigned due to the poor differentiation of certain tumor cells. Pleomorphic cell carcinoma, large cell carcinoma, large cell neuroendocrine carcinoma and adenosquamous carcinoma are classified as poorly-differentiated tumors. In the present study, these tumors were found to be particularly likely to be pre-operatively diagnosed as NOS. Pleomorphic carcinoma accounted for 12.6% of the cases in the NOS group, even though the true prevalence of pleomorphic carcinoma has been reported to be only 1.6% (16). Due to their heterogeneity and poorly-differentiated tumor cells, these tumor types are difficult to diagnose on pre-operative pathological examination. Consequently, resected specimens were necessary to achieve definitive diagnoses. Additionally, these subtypes are associated with a poor prognosis even if the disease is diagnosed at early stages and resected (16,17). The poor prognosis of the NOS group in the present series appears to be affected by the characteristics of these tumor cells.

It has been reported that sublobar resection, including segmentectomy and wedge resection, is not inferior to lobectomy for patients with small-sized NSCLC. Studies by Okada *et al* (18,19) indicated that sublobar resection should be considered as an alternative surgical option for stage IA NSCLC tumors that are ≤2 cm in size, even for low-risk patients. Conversely, in the case of certain aggressive tumors, sublobar resection may be inappropriate for curative surgery. Indeed, Varlotto *et al* (20) showed that, among patients with stage I NSCLC, sublobar resection is associated with a greater risk of local recurrence than lobectomy, particularly for patients with poorly-differentiated tumors or tumors of >2 cm in size. Hattori *et al* (6) showed that sublobar resection is not feasible for purely solid tumors, particularly those with a high maximum standardized uptake value, including small lung cancers. The present results indicate that the NOS classification is associated with poor survival, even for stage I cases. Moreover, the pathological diagnosis of the resected specimens indicated that poorly-differentiated tumors, such as pleomorphic cell carcinoma, are significantly more frequent in NOS patients; a finding that is concordant with the poor prognosis observed for these patients. Therefore, NOS cases may not be good candidates for sublobar resection.

In the present study, 88 cases (58.3%) were diagnosed on the basis of cytomorphology alone and the remaining 63 cases were evaluated histologically. Recent clinical observations of patients with advanced lung cancer have motivated pathologists to exert the additional effort that is necessary to distinguish between the histological subtypes, improving the overall quality of subtyping. In comparison, cytological diagnoses of squamous and non-squamous lung cancer subtypes have only a fair degree of accuracy (21). Moreover, independent pathological review is not available to all oncologists in daily practice, limiting the further subclassification of NSCLC following the initial diagnosis. Several recommendations for the pre-operative evaluation of patients with resectable NSCLC do not indicate definitive pre-operative histological subtyping (22). In the present study, the prognosis did not depend on the mode of NOS diagnosis (cytological or histological), indicating that pre-operative NOS had the role of a prognostic factor regardless of the two differing diagnostic modes.

There are certain limitations to the present study. First, the study data was analyzed retrospectively and without central pathological review, although all diagnoses were reviewed by two expert pathologists. Second, although sublobar resection may be inappropriate for curative surgery in the early stage of NOS cases, the prognoses of the NOS patients undergoing sublobar resection was not evaluated due to the small sample sizes. This matter should be formally investigated and discussed in a larger population in the future.

In conclusion, the present study found that pre-operative NOS diagnosis was associated with significantly poorer survival among patients with NSCLC, even those with stage I disease. In conjunction with other clinicopathological parameters, NOS can be a useful prognostic factor when selecting a treatment strategy for NSCLC.

## Figures and Tables

**Table I:** Clinicosurgical characteristics of the study population.

Factor	NOS group	Confirmed group	P-value
Total, n	151	1762	
Gender, n (M/F)	127/24	1194/568	<0.001
Age, years (mean ± SD)	69±9	68±9	0.453
BMI (mean ± SD)	22.4±2.8	22.3±3.0	0.521
Smoking status, n			<0.001
Smoker	130	1211	
Non-smoker	21	551	
FEV1.0, liters (mean ± SD)	2.22±0.60	2.19±0.61	0.090
FEV1.0/FVC, % (mean ± SD)	69.8±12.6	73.0±10.6	0.020
Size of tumor, mm (mean ± SD)	35.3±16.0	33.9±16.6	0.410
Procedure, n			0.159
Pneumonectomy	0	15	
Lobectomy	120	1353	
+ extended resection	14	116	
Segmentectomy	12	197	
Wedge resection	5	81	
Adjuvant chemotherapy, n (yes/no)	57/94	502/1260	0.003

FEV1.0, forced expiratory volume in 1 second; FVC, forced vital capacity; BMI, body mass index; NOS, not otherwise specified; SD, standard deviation.

**Table II:** Histopathological characteristics.

Factor	NOS group	Confirmed group	P-value
Total, n	151	1762	
Pathological stage, n (%)			
IA	39 (25.8)	604 (34.3)	0.127
IB	51 (33.8)	479 (27.1)	
IIA	24 (15.9)	270 (15.3)	
IIB	14 (9.3)	141 (8.0)	
IIIA	23 (15.2)	249 (14.1)	
IIIB	0 (0.0)	9 (0.5)	
IV	0 (0.0)	10 (0.6)	
Vessel invasion, n (Yes/no)	99/52	965/797	<0.001
Lymphatic invasion, n (Yes/no)	56/95	721/1041	0.350
Pleural invasion, n (P0/P1/P2/P3)	88/37/7/19	1149/330/135/148	0.053
Histology, n (%)			
Adenocarcinoma	60 (39.7)	1144 (64.9)	<0.001
Squamous cell carcinoma	42 (27.8)	481 (27.3)	0.969
Adenosquamous carcinoma	8 (5.3)	34 (1.9)	0.014
Large cell carcinoma	8 (5.3)	29 (1.6)	0.019
Large cell neuroendocrine carcinoma	12 (7.9)	47 (2.7)	0.002
Pleomorphic cell carcinoma	19 (12.6)	23 (1.3)	<0.001
Sarcomatoid carcinoma	2 (1.3)	4 (0.2)	0.074

NOS, not otherwise specified.

**Table III:** Univariate and multivariate analyses of factors associated with prognosis.

Factor	Hazard ratio	P-value
Univariate analysis		
NOS (Yes vs. no)	1.47 (1.07–1.96)	0.016
Age, years (≥65 vs. <65)	1.39 (1.16–1.68)	<0.001
Gender (male vs. female)	2.01 (1.64–2.49)	<0.001
BMI (≥22 vs. <22)	0.78 (0.66–0.93)	0.005
P-stage (I vs. II–IV)	3.00 (2.53–3.57)	<0.001
Surgical procedure (non-limited vs. limited)	1.22 (0.97–1.55)	0.080
COPD (FEV1.0% ≤70 vs. >70)	1.40 (1.18–1.66)	<0.001
Smoking status (Yes vs. no)	1.81 (1.49–2.21)	<0.001
Histology (Sq vs. non-Sq)	1.66 (1.39–1.98)	<0.001
Vessel invasion (Yes vs. no)	2.17 (1.83–2.58)	<0.001
Lymphatic invasion (Yes vs. no)	2.36 (1.98–2.79)	<0.001
Pleural invasion (P1–3 vs. P0)	2.25 (1.85–2.73)	<0.001
Adjuvant chemotherapy (Yes vs. no)	0.86 (0.70–1.04)	0.140
Multivariate analysis		
NOS (Yes vs. no)	1.40 (1.02–1.86)	0.041
Age, years (≥65 vs. <65)	1.55 (1.28–1.88)	<0.001
Gender (male vs. female)	1.51 (1.16–1.96)	0.002
BMI (≥22 vs. <22)	0.82 (0.69–0.97)	0.025
P-stage (I vs. II–IV)	2.22 (1.84–2.69)	<0.001
COPD (FEV1.0% ≤70 vs. >70)	1.02 (0.84–1.23)	0.818
Smoking status (Yes vs. no)	1.11 (0.86–1.45)	0.409
Histology (Sq vs. non-Sq)	1.23 (1.02–1.49)	0.028
Vessel invasion (Yes vs. no)	1.27 (1.06–1.56)	0.012
Lymphatic invasion (Yes vs. no)	1.55 (1.28–1.88)	<0.001
Pleural invasion (P1–3 vs. P0)	1.24 (1.02–1.49)	0.041

NOS, not otherwise specified; FEV1.0, forced expiratory volume in 1 sec; BMI, body mass index; P-stage, pathological stage; COPD, chronic obstructive pulmonary disease; Sq, squamous cell carcinoma.
